# Utility of blood as the clinical specimen for the diagnosis of ocular toxoplasmosis using uracil DNA glycosylase-supplemented loop-mediated isothermal amplification and real-time polymerase chain reaction assays based on REP-529 sequence and B1 gene

**DOI:** 10.1186/s12879-022-07073-3

**Published:** 2022-01-25

**Authors:** Bahman Rahimi Esboei, Shirzad Fallahi, Mohammad Zarei, Bahram Kazemi, Mehdi Mohebali, Saeedeh Shojaee, Parisa Mousavi, Aref Teimouri, Raziyeh Mahmoudzadeh, Mirataollah Salabati, Hossein Keshavarz Valian

**Affiliations:** 1 Department of Parasitology and Mycology, School of Medicine, Tonekabon Branch, Islamic Azad University, Tonekabon, Iran; 2 Department of Medical Parasitology and Mycology, Lorestan University of Medical Sciences, Khorramabad, Iran; 3 Retina Service, Farabi Eye Hospital, Tehran University of Medical Sciences, Tehran, Iran; 4 Cellular and Molecular Biology Research Center, Shahid Beheshti University of Medical Sciences, Tehran, Iran; 5 Department of Medical Parasitology and Mycology, School of Public Health, Tehran University of Medical Sciences, Tehran, Iran; 6 Skin Diseases and Leishmaniasis Research Center, Isfahan University of Medical Sciences, Isfahan, Iran; 7 Department of Parasitology and Mycology, School of Medicine, Shiraz University of Medical Sciences, Shiraz, Iran; 8 Wills Eye Hospital, Mid Atlantic Retina, Thomas Jefferson University, Philadelphia, PA USA; 9 Center for Research of Endemic Parasites of Iran, Tehran University of Medical Sciences, Tehran, Iran

**Keywords:** *Toxoplasma gondii*, Chorioretinitis, UDG-LAMP, Real-time PCR, Peripheral blood

## Abstract

**Background:**

Ocular infection with *Toxoplasma gondii* is a major preventable cause of blindness, especially in young people. The aim of the present study was to assess detection rate of *T. gondii* DNA in blood samples of clinically diagnosed of ocular toxoplasmosis using uracil DNA glycosylase-supplemented loop-mediated isothermal amplification (UDG-LAMP) and real-time quantitative PCR (qPCR) based on REP-529 and B1.

**Methods:**

One hundred and seventeen patients with clinically diagnosed ocular toxoplasmosis (OT) were participated in the study as well as 200 control patients. Peripheral blood samples were assessed using UDG-LAMP and qPCR techniques targeting REP-529 and B1.

**Results:**

Detection limits of qPCR using REP-529 and B1 were estimated as 0.1 and 1 fg of *T. gondii* genomic DNA, respectively. The limits of detection for UDG-LAMP using REP-529 and B1 were 1 and 100 fg, respectively. In this study, 18 and 16 patients were positive in qPCR using REP-529 and B1, respectively. Based on the results of UDG-LAMP, 15 and 14 patients were positive using REP-529 and B1, respectively. Results of the study on patients with active ocular lesion showed that sensitivity of REP-529 and BI targets included 64 and 63%, respectively using qPCR. Sensitivity of 62 and 61%, were concluded from UDG-LAMP using REP-529 and B1 in the blood cases of active ocular lesion. qPCR was more sensitive than UDG-LAMP for the detection of *Toxoplasma gondii* DNA in peripheral blood samples of patients with clinically diagnosed toxoplasmic chorioretinitis. Furthermore, the REP-529 included a better detection rate for the diagnosis of ocular toxoplasmosis in blood samples, compared to that the B1 gene did. Moreover, the qPCR and UDG-LAMP specificity assessments have demonstrated no amplifications of DNAs extracted from other microorganisms based on REP-529 and B1.

**Conclusions:**

Data from the current study suggest that qPCR and UDG-LAMP based on the REP-529 are promising diagnostic methods for the diagnosis of ocular toxoplasmosis in blood samples of patients with active chorioretinal lesions.

## Background

Ocular toxoplasmosis (OT), one of the major causes of posterior uveitis globally, can lead to vision-threatening complications such as retinal detachment, choroidal neovascularization and glaucoma. The disease is associated with congenital or postnatally acquired infection caused by the ubiquitous apicomplexan parasite *Toxoplasma gondii* (*T. gondii*) and classically presents as a necrotizing retinochoroiditis that can be single [1], multiple [2] or satellite [3] to atrophic-pigmented scars. Congenitally infected people, who are asymptomatic at birth [4], may later develop toxoplasmosis symptoms [5]. Diagnosis of OT is routinely carried out through ophthalmic examinations and various clinical findings that confirm *T. gondii* infection of the retinochoroiditis. However, the clinical presentation can at times prove to be misleading [6, 7], requiring further biological tests [8] to be either confirmed or refuted [9]. When definitive clinical diagnosis cannot be carried out, a direct detection of *T. gondii* DNA using conventional polymerase chain reaction (PCR) [10] and antibody detection [11] with titer interpretation from the blood and/or ocular samples are successfully used to verify the primary diagnosis [12–14]. These methods cannot only confirm the OT diagnosis but can also rule out other similar infectious diseases [7, 15].

From various molecular techniques, real-time quantitative PCR (qPCR) and LAMP assays are particularly popular because of their high sensitivity, specificity and speed. The LAMP technique is carried out under isothermal reaction conditions and does not need advanced equipment [16–19]. The major problem of using molecular methods for the ocular fluids is linked to the invasive nature of these methods [14]. Significantly, peripheral blood sampling is less invasive than ocular fluid sampling. In the current study, qPCR and UDG-LAMP assays were assessed for the detection of *T. gondii* DNA in blood samples of clinically diagnosed OT patients using repetitive REP-529 sequence and B1 gene.

## Methods

### Patients and clinical sample collections

**Fig. 1:**
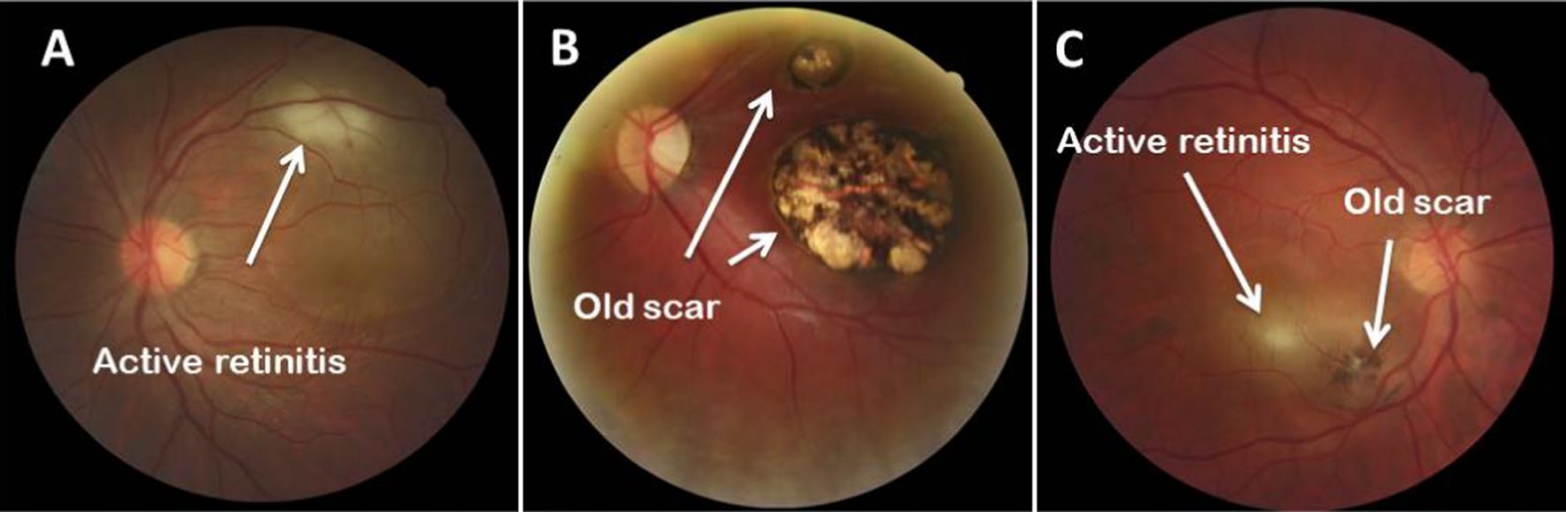
Clinical manifestation of ocular toxoplasmosis; **A** *Toxoplasma* retinitis (active disease), **B** Retinochoroiditis scar (old scar), **C** *Toxoplasma* retinitis and adjacent retinochoroiditis scar (reactivated disease)

This cross-sectional study was designed using 117 immunocompetent patients with clinically diagnosed OT in Farabi Eye Hospital, Tehran, Iran, from October 2015 to August 2017. The OT was clinically diagnosed by a retina specialist (MZ). Chorioretinal involvements of *T. gondii* were classified into three major groups based on fundoscopic examination, including (1) Group A (active lesion): an area of white edematous retina with ill-defined borders and overlying vitritis, usually accompanied by nearby vasculitis (Fig. 1A). This appearance represents the active form of disease and when seen with no scars is considered as a consequence of the primary acquired infection [20]; (2) Group B (old scar): a chorioretinal scar with sharp borders and variable degrees of dark pigmentation (Fig. 1B). This appearance represents the inactive form of disease and is considered as a reminiscence of the active prior chorioretinitis [21, 22] and (3) Group C (reactivated disease): a combination of the two forms, including an area of active chorioretinitis in eyes with toxoplasmic chorioretinal scars, usually close to an active chorioretinitis focus (Fig. 1C). This form may be resulted from the failure of immune system to continually limit the infected foci and subsequent release of the tissue cyst content into the close tissues [23]. Moreover, 200 patients with other eye diseases other than toxoplasmosis, including viral keratitis (39 samples), amebic keratitis (38 samples), fungal keratitis (42 samples), bacterial endophthalmitis (64 samples) and cataract (17 samples) were participated as controls. Blood samples (up to 3.5 mL) were collected from the participants using EDTA-containing tubes. These blood samples have been verified using various biological techniques by the authors in previous studies [24, 25].

### DNA extraction

DNA was extracted from the whole blood using QIAamp Genomic DNA Blood Mini Extraction Kit (Qiagen, Hilden, Germany) based on the manufacturer instructions and kept frozen at − 20 °C for further use in qPCR and UDG-LAMP reactions [25]. The reference DNA was extracted from a virulent RH strain of *T. gondii* (Type I), which was previously collected from peritoneal cavity of the infected mice [26].

### qPCR

A TaqMan-probe qPCR targeting REP-529 and B1 was developed using StepOne Real-Time PCR System (Applied Biosystems, Foster City, CA, USA) with 45 cycles of amplification. REP-529 is a highly repetitive sequence with 200–300 copies in *T. gondii* genome [27]. The B1 gene has 35 copies in the genome and is conserved indifferent parasite strains [28]. The PCR reaction included RealQ Plus Master Mix for probes labeled with reporter fluorophor 6-carboxyfluorescein (Ampliqon, Odense, Denmark) (10 µL), TagMan probe (FAM-CCCTCGCCCTCTTCTCCACTCTTCAA-3-BHQ1) (0.2 µM), extracted DNA (5 µL), forward (5ʹ-CTTCGTCCAAGCCTCCGA-3ʹ) and reverse (5ʹ-GACGCTTTCCTCGTG GTGAT-3ʹ) primers (0.4 µL) and distilled water (4 µL). The PCR thermal cycling was carried out at 95 °C for 5 min and continued for 40 cycles of 95 °C for 1 min, annealing at 55 °C for 1 min and extension at 72 °C for 1 min. The *gapdh* gene was used as housekeeping gene with primers common to all mammalian species to check the quality of DNA [18]. An internal control (TaqMan exogenous positive control) was used, and each run was considered valid if the positive, negative, and internal controls were acceptable. The result was considered negative if repeated atypical amplification curves with proper amplification of the internal control was present. A sample was considered inhibited if amplification of the internal control failed and there was no amplification or atypical amplification curve for the target of interest.

### Uracil DNA glycosylase-supplemented loop-mediated isothermal amplification assay

**Fig. 2:**
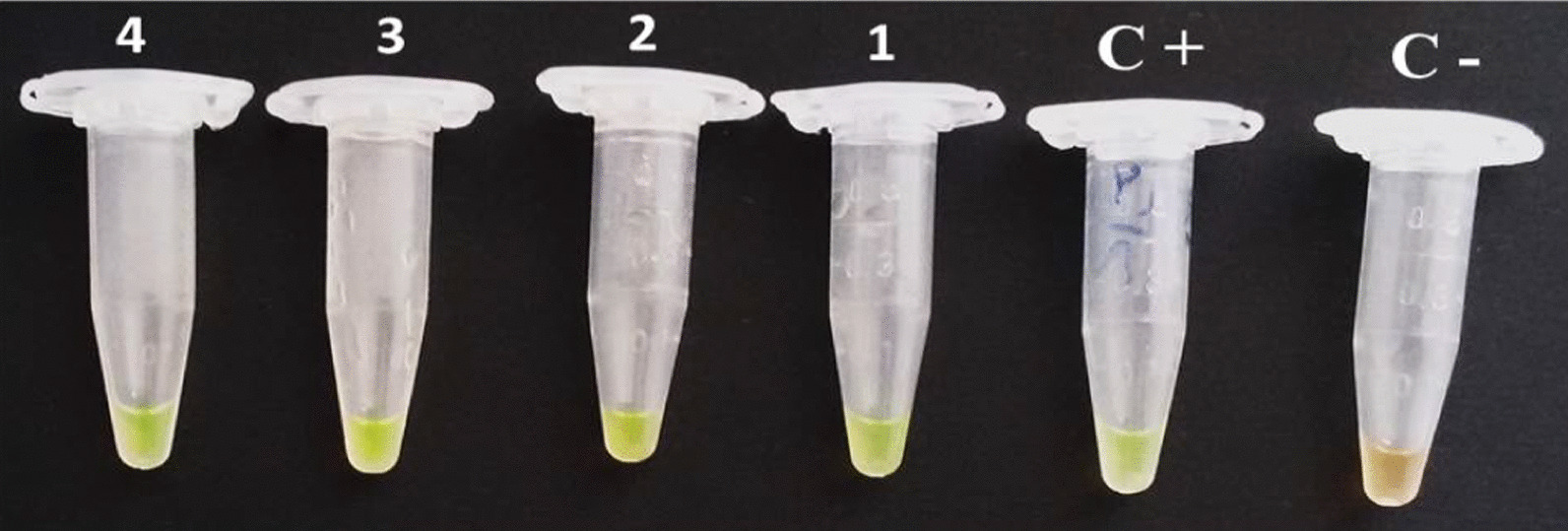
Tubes with UDG-LAMP reaction stained with SYBR Green I. C+ , positive control; C-, negative control; 1–4, different patients with positive reactions. The color changes from orange (negative reaction) to green (positive reaction)

UDG-LAMP assay was successfully performed in our laboratory as described previously [29]. Specific oligonucleotide primers for *T. gondii* used for the LAMP assay were designed based on REP-529 and B1 regions of the parasite genome (Table 1). The total reaction master mix volume was 25  μL consisting of 1 μL of template DNA, 40 picomol of each of FIP and BIP primers, 20 pmol of each LF and LB primers (used only in REP-LAMP), 5 pmol of each of F3 and B3 primers, 8 U of Bst2 DNA polymerase (New England Biolabs, USA), 1.4 mmol/L of deoxyuridine triphosphates (dUTP) instead of dTTP and 2X reaction buffer, containing 1.6 mol/L betaine (Sigma Aldrich, USA), 40 mmol/L Tris–HCl (pH 8.8), 20 mmol/L of KCL, 20 mmol/L of (NH4)2SO4, 16 mmol/L of MgSO4 and 0.2% tween 20). The LAMP assay were carried out at 60–67 °C for 30, 45, 60 and 75 min to find the optimum time and temperature conditions and finally; 63 °C and 60 min were the best temperature and time for both target genes, respectively. Addition of 3 µL of the fluorescent detection reagent, diluted SYBR Green I (Invitrogen, Carlsbad, California, USA), to the reaction tube after LAMP reaction enables visible analysis of the results under daylight and/or UV light. The positive amplification was read through observation of change in color of the reaction mixture following addition of dye to the tube and the color changes from orange (negative reaction) to green (positive reaction) (Fig. 2). The results were further verified by electrophoresis of LAMP products in 1.5% agarose gel electrophoresis stained with DNA Safe Stain (SinaClon, Iran), and visualized under UV light. To prevent self-amplification of the primers, reactions were carried out in the absence of DNA templates. The *T. gondii* RH-strain DNA and double distilled water were used as positive and no template controls, respectively. To assess reproducibility of the assay, all experiments were carried out in duplicate. The protocol is fully described in a previous study by the authors [29, 30].

**Table 1:** Nucleotide sequences of LAMP primers for molecular diagnosis of *T. gondii*

Genes	Primers	Size	Accession number
B1	BIP-5′-TCGCAACGGAGTTCTTCCCAGTTTTGGCCTGATATTACGACGGAC-3′	212 bp	AF179871
	FIP-5′-TGACGCCTTTAGCACATCTGGT TTTTGATGCTCAAAGTCGACCGC-3′		
	F3-5′-GGGAGCAAGAGTTGGGACTA-3′		
	B3-5′CAGACAGCGAACAGAACAGA-3′		
REP-529	BIP-5′-TGGTTGGGAAGCGACGAGAGTTCCAGGAAAAGCAGCCAAG-3′	202 bp	AF146527
	FIP-5′-TCCTCACCCTCGCCTTCATCTAGGACTACAGACGCGATGC-3′		
	LF-5′-TCCAAGACGGCTGGAGGAG-3′		
	LB-5′-CGGAGAGGGAGAAGATGTTTCC-3′		
	F3-5′-CCACAGAAGGGACAGAAGTC-3′		
	B3-5′-TCCGGTGTCTCTTTTTCCAC-3′

### Analytical sensitivity and specificity of qPCR and UDG-LAMP assays

To assess analytical sensitivity of the qPCR and UDG-LAMP techniques for the detection of *T. gondii* (RH strain) DNA, tenfold serial dilutions of *T. gondii* DNA with 1 pg to 0.01 fg concentrations were prepared and used in qPCR and LAMP assays targeting REP-529 and B1. The construction is performed with various concentrations of DNA. Next, the cycle threshold (CT) values were plotted as mean (triplicate) against the standard curve values to determine the detection limit of both primer sets. Parasite concentrations are determined after the calculation of the linear regression equation (y = ax + b), where y = CT; a = curve slope (slope); x = parasite number; and b = where the curve intersects y-axis (y intercept) [31, 32].

To assess the analytical specificity of the qPCR and UDG-LAMP techniques, extracted DNAs from other microorganisms, including *Leishmania tropica, L. major, Giardia lamblia, Cryptosporidium parvum, Entamoeba dispar, Trichostrongylus colubriformis, Corynosoma capsicum*, *Candida albicans* and *Acanthamoeba* spp. as well as human chromosomal DNA, were used as templates in qPCR and LAMP assays using specific primers of REP-529 and B1.

### Statistical analysis

Data were analyzed using SPSS Software v.21 (SPSS Inc. Chicago, IL, USA). The χ^2^ test was used to calculate diagnostic sensitivity and specificity of the qPCR and UDG-LAMP techniques. Agreements between the clinical and molecular assays were demonstrated using Cohen’s kappa coefficient [33]. Linear regressions were constructed from the standard curves for REP-529 and B1 and CT mean comparison of both primer pairs. Both curves were statistically analyzed using an unequal-variance t-test based on a critical value of p ≤ 0.05.

## Results

### Distribution of participants

Of the total samples, 55.55% (65/117) belonged to males and 44.44% (52/117) to females. Thirty six (30.76%) patients were up to 10 years old, 50 (42.73%) patients were 11–20 years old, 23 (19.65%) patients were 21–30 years old and eight (6.83%) patients were 31–40 years old. Of 117 patients, 37 (31.62%) patients included active lesions (Group A), 13 (11.11%) patients included pigmented scars (Group B) and 67 (57.26%) patients included pigmented scars with active lesions (Group C).

### qPCR and UDG-LAMP of the clinical samples

**Fig. 3:**
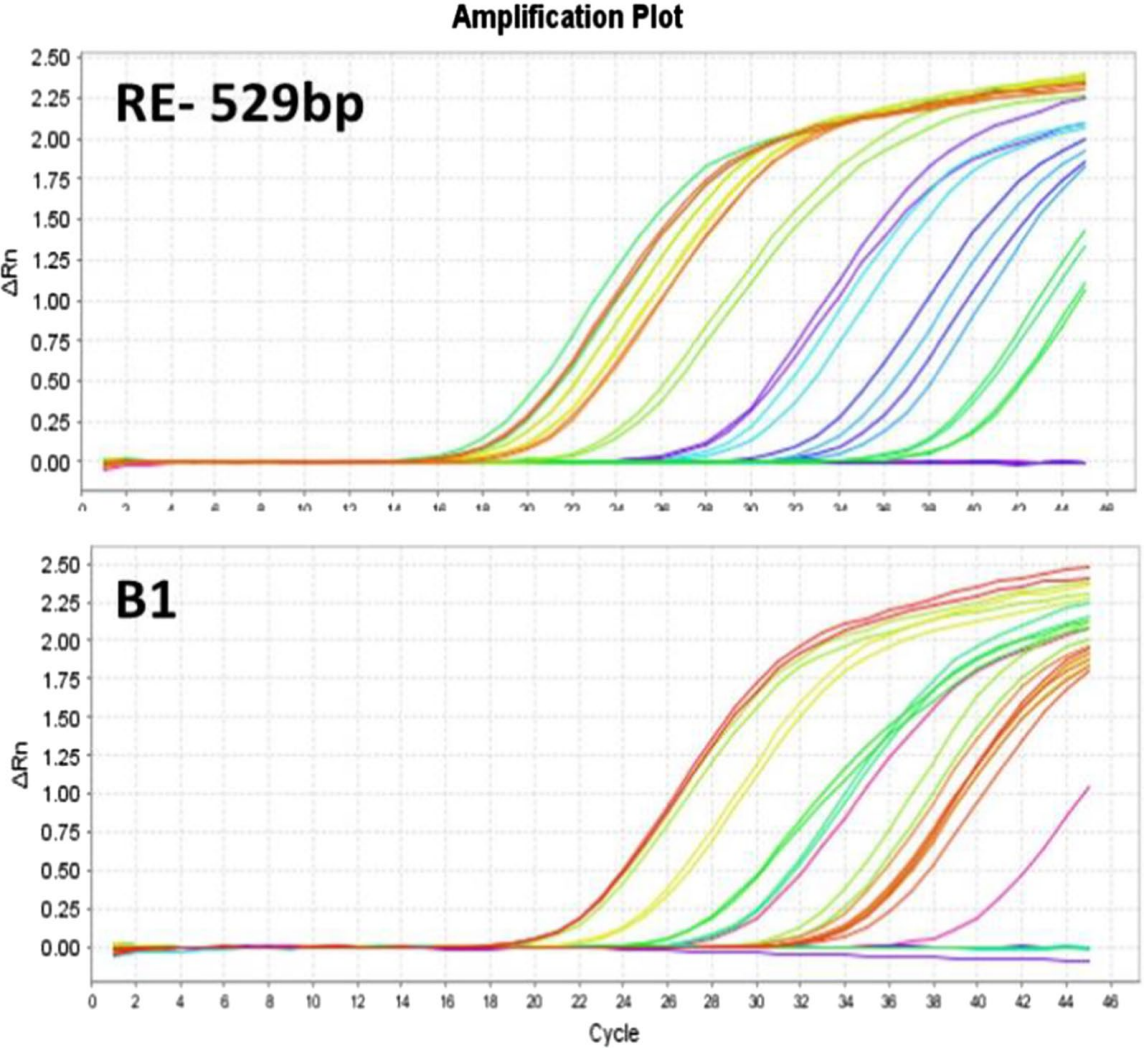
qPCR amplification plot based on REP-529 and B1 targets of *T. gondii* from blood samples of patients suspected to ocular toxoplasmosis

Out of 37 patients in Group A (patients with active lesions), 18 and 16 patients were positive by qPCR using REP-529 and B1 and 15 and 14 patients were positive by UDG-LAMP using REP-529 and B1, respectively. All positive results using UDG-LAMP had 100% overlaps with positive results of qPCR using REP-529 and B1. None of the patients in other clinical groups (Groups B and C) were positive using molecular assays (Table 2). All patients in control group were negative for REP-529 and B1 using qPCR and UDG-LAMP. Amplification plot and derivative melt curve analysis of the *T. gondii* based on REP-529 and B1 targets are shown in Fig. 3. The results of chi-square test indicated that qPCR method based on REP-529 was the most sensitive than UDG-LAMP using REP-529 and B1 (*P* = 0.003).

**Table 2:** Summary results of ocular toxoplasmosis using qPCR and LAMP techniques based on REP-529 sequence and B1 gene among different clinically manifestations

Molecular and clinical results	Real-time PCR		LAMP	
	REP-529	B1	REP-529	B1
Group A (n = 37)	18 (48.64%)	16 (43.24%)	15 (40.54%)	14 (37.83%)
Kappa value	0.796	0.732	0.664	0.628
Group C (n = 67)	0 (0.0%)	0 (0.0%)	0 (0.0%)	0 (0.0%)
Kappa value	0.558	0.584	0.611	0.639
Group B (n = 13)	0 (0.0%)	0 (0.0%)	0 (0.0%)	0 (0.0%)
Kappa value	0.122	0.136	0.148	0.154
*P*- value	0.001	0.001	0.001	0.001
Sensitivity	64%	63%	62%	61%
Specificity	100%	100%	100%	100%
Positive predictive value (PPV)	100%	100%	100%	100%
Negative predictive value (NPV)	90%	90%	90%	89%

Group A, 37 cases of active lesion (*Toxoplasma* retinitis); Group B, 67 cases of old scar (Retinochoroiditis scar); Group C, 13 cases of reactivated disease (*Toxoplasma* retinitis and adjacent retinochoroiditis scar)

### Analytical sensitivity of qPCR and UDG-LAMP assays

**Fig. 4:**
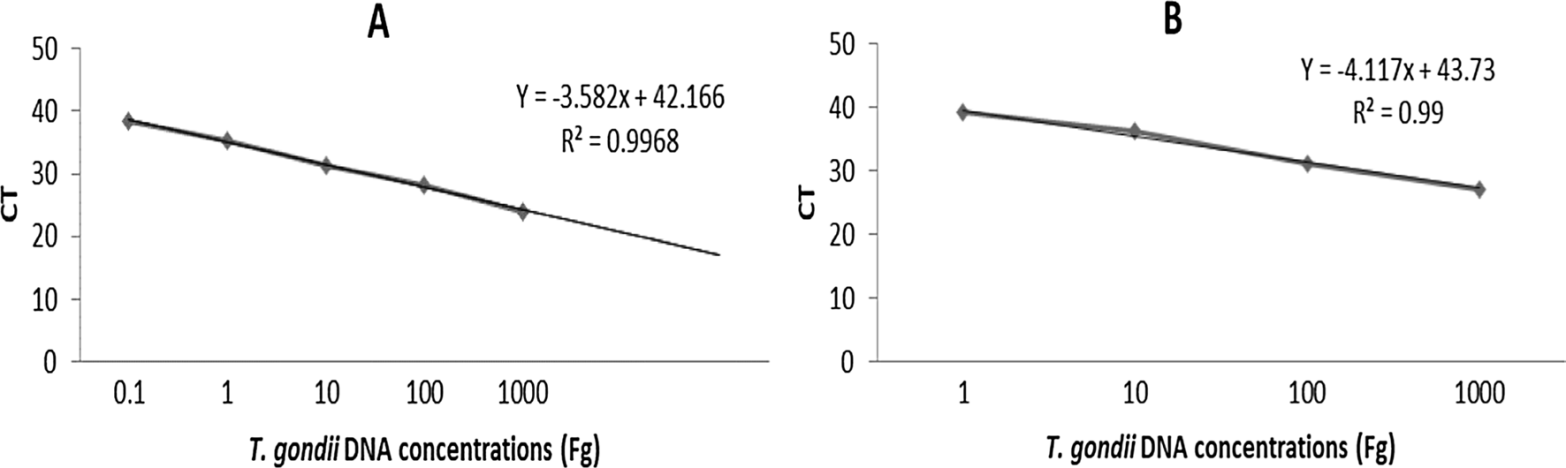
Standard curve of *T. gondii*, tachyzoites using REP-529 (**A**) and B1 (**B**) primer sets, respectively. Results are shown as mean cycle threshold (CT) obtained from triplicate of each DNA concentration. Standard curve analysis was performed in tenfold serial dilutions of *T. gondii* DNA extracted from tachyzoites, at initial concentration of 1000 fg/μL

**Fig. 5:**
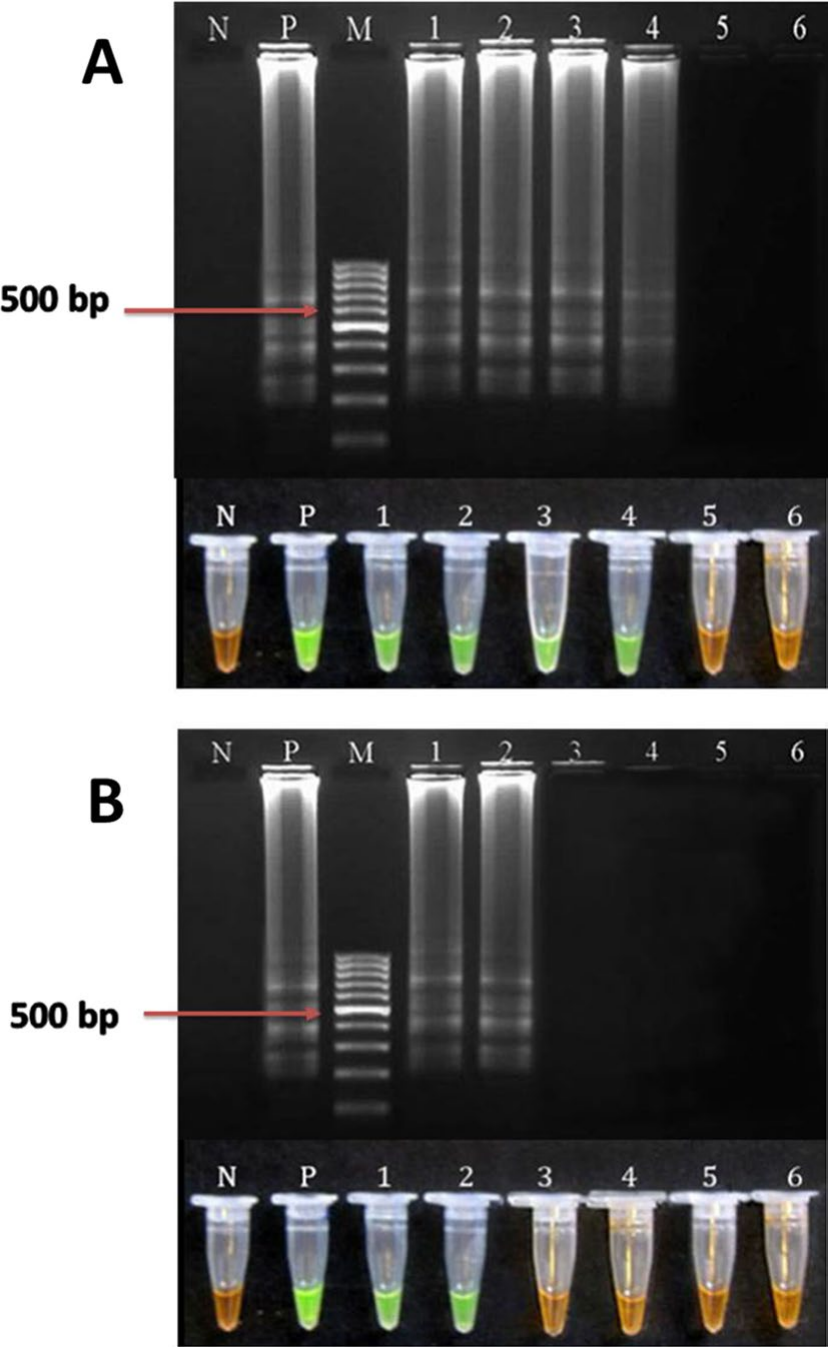
Comparison of the sensitivity of the REP-529 bp (**A**) and B1 (**B**) genes using UDG-LAMP assay in diagnosis of *T. gondii* (RH strain) DNA. N: negative control, P: positive control, M: 100 bp DNA Marker, lanes 1–6: 1 pg, 100 fg, 10 fg, 1 fg, 0.1 fg and 0.01 fg of *T. gondii* (RH strain) DNA

The first step was to assess the reportable range of the reaction that was determined utilizing *T. gondii* DNA isolate from tachyzoites (RH strain) using the REP-529 (Fig. 4A) and B1 (Fig. 4B). The current results showed that the detection limits for UDG-LAMP using REP-529 and B1 were 1 and 100 fg, respectively. Detection limits of qPCR using REP-529 and B1 were estimated as 0.1 and 1 fg of *T. gondii* genomic DNA, respectively (Figs. 4, 5).

### Specificity of qPCR and UDG-LAMP assays

**Fig. 6:**
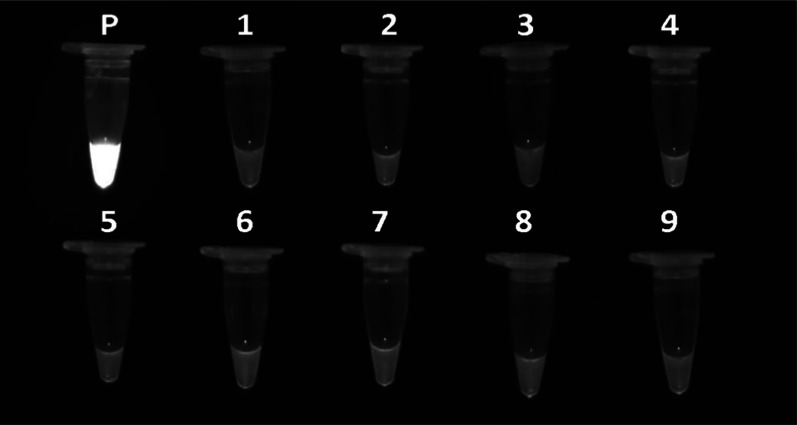
Analytical specificity of UDG-LAMP assay. P: positive control (*T. gondii*, RH strain), lanes 1–9: DNA of *Giardia lamblia, Cryptosporidium parvum, Entamoeba dispar, Leishmania major, Acanthamoeba, Trichostrongylus colubriformis, Corynosoma capsicum, Candida albicans* and Human Genomic DNA

Using qPCR and UDG-LAMP, no amplifications were detected in reactions with DNA of *G. lamblia, C. parvum, E. dispar, L. major, Acanthamoeba* spp.*, T. colubriformis, C. capsicum* and *C. albicans* as well as human genomic DNA (Fig. 6).

## Discussion

Although clinical examination is the standard method for the diagnosis of OT in the second and third forms of the disease (old scars and reactivated disease), the first form of disease (active lesions) cannot always be differentiated from other chorioretinal inflammations based on funduscopic appearance alone [34]. Based on the previous studies, ocular fluid samples are the most sensitive sources for the molecular diagnosis of OT [15, 34, 35]. Molecular examination of the aqueous humor puncture allows identification of coinfections or various etiological agents for the infectious uveitis. It is noteworthy that coinfections need various treatments in addition to anti-*Toxoplasma* therapy [15]. However, the most important limitation of this method is linked to its invasive nature that may hurt the eyes [20, 36].

PCR of blood samples from patients with OT produced similar results to those from PCR of aqueous humor samples, avoiding problems associated with ocular puncture [37]. More recently, Khanaliha et al. have reported that peripheral blood mononuclear cells (PBMCs) are appropriate for the assessment of toxoplasmic chorioretinitis. Furthermore they have reported that PCR with bradyzoite genes is useful for the diagnosis of toxoplasmic chorioretinitis in these cells [38]. In a previous study by the current authors, agreements between the two approaches of nested PCR and serological assays were assessed and results highlighted that nested-PCR of peripheral blood samples was a useful minimally invasive technique for providing direct evidence of the presence of *T. gondii* in patients with OT, especially in recently acquired infections. Nested-PCR with REP-529 target included 57 and 100% sensitivity and specificity in diagnosis of recently acquired OT, respectively [25]. In contrast, studies have revealed that molecular testing on peripheral blood samples is not sufficiently sensitive for the detection of *T. gondii* in patients with OT [35]. In a study, Bourdin et al. reported 35.9% sensitivity in diagnosis of OT using PCR of peripheral blood samples. These studies showed that in OT it is possible to detect *T. gondii* DNA in peripheral blood samples without a direct relation of *Toxoplasma* activity within the eye. They concluded that PCR technique was not sensitive enough for the diagnosis of OT from blood samples [39]. Several factors affect sensitivity of molecular techniques, including volumes and types of the samples, DNA markers, gene targets and types of the molecular methods [34].

In the current study, we detected *T. gondii* DNA in the blood of patients with active ocular lesions (Group A) using qPCR and UDG-LAMP based on REP-529 and B1, but not in the blood of patients with old scars (Group B) and reactivated disease (Group C), which suggests that parasitaemia is associated with ongoing disease. Tachyzoites of *T. gondii* have been isolated from the blood of immunocompetent patients with the ocular disease but not from the blood of patients with recurrent retinochoroiditis [40]. However, Silveira et al. have reported that subclinical parasitaemia is present in patients with either acute or chronic toxoplasmosis, regardless of the presence of ocular disease [41]. Although assays of this study did not include high sensitivities in all OT patients, these sensitivities were acceptably high in patients from the Group A. The presence of an old pigmented scar is usually the most useful and characteristic clinical finding in the diagnosis of OT. This clinical finding is absent in patients with active lesions resulted from recently acquired toxoplasmosis in contrast to patients with active lesions resulted from reactivation of congenitally acquired toxoplasmosis (Group C) [2, 22]. It is noteworthy that the Group B patients included old inactive scars alone with no active chorioretinitis lesions and hence did not need treatments and Group C patients included pathognomonic clinical findings of simultaneous presence of old scars and active chorioretinitis lesions and thus did not need confirmatory paraclinical diagnostic assessments [21, 23].

In the current study, detection limits of qPCR using REP-529 and B1 targets were respectively estimated as 0.1 and 1 fg of the *T. gondii* genomic DNA. Detection limits of UDG-LAMP using the highlighted targets were 1 and 100 fg of the *T. gondii* genomic DNA, respectively. Kong et al. reported detection limits of RE-LAMP, B1-LAMP and RE-nested PCR assays as 0.6, 60 and 600 fg of the DNA templates, respectively [42]. A similar study by Zhang et al. revealed that the detection limits of LAMP and conventional PCR assays using REP-529 target were 1 and 10 pg, respectively [43]. Various target genes such as SAG1, SAG2, SAG3, SAG4, ITS, B1, REP-529, P30, GRA4, GRA6 and GRA7 have been used to detect toxoplasmosis [44–48]. The results have shown that both REP-529 and B1 are highly sensitive; REP-529 is more sensitive than B1 gene. [32, 49, 50]. Therefore, the present technique was based on the REP-529 and B1. Based on the results from a study by da Silva et al., it has been shown that the REP-529 includes 200–300 copies in the genome of *Toxoplasma* parasite, the highest copy number within all studied genes that are quite specific to *T. gondii* [51]. Furthermore, several studies have reported that the B1 gene includes 35 copies in genome of *T. gondii*, which is completely specific [17, 52–54]. The SAG1, SAG2, SAG3, SAG4, GRA4, GRA6 and GRA7 genes include only one copy in genome of *T. gondii*, which are specific as well [46]. Studies have shown that the number of copies of each gene fragment includes direct relationships with the diagnosis sensitivity [48, 52]. Another factor that is important in selecting appropriate gene fragments is specificity [55]. The current results have shown no cross-reactivity with DNA corresponding to parasitic infections other than toxoplasmosis using qPCR and UDG-LAMP based on REP-529 and B1, thereby insuring high specificity for target amplification [27, 56].

## Conclusion

Results of the current study have shown that samples from peripheral blood samples can potentially be used for the molecular diagnosis of OT in patients with active chorioretinal lesions. In the present study, qPCR was more sensitive than UDG-LAMP. However, UDG-LAMP can be used in fields, potentially excluding needs of expensive PCR machines and gel electrophoresis devices as well as laborious DNA extraction processes. Regarding the gene target, RE was more sensitive than B1 gene. Moreover, the qPCR and UDG-LAMP specificity assessments have demonstrated no amplifications of DNAs extracted from other microorganisms based on REP-529 and B1. Therefore, data from the current study suggest qPCR and UDG-LAMP of REP-529 as promising diagnostic techniques for the diagnosis of OT in blood samples of patients with active chorioretinal lesions.

## Data Availability

All data generated or analyzed during this study are included in this published article. The raw data are available from the corresponding author on reasonable request.

## Abbreviations

OT: Ocular toxoplasmosis
UDG-LAMP: Uracil DNA glycosylase-supplemented loop-mediated isothermal amplification
PCR: Polymerase chain reaction
EDTA: Ethylenediaminetetraacetic acid
SPSS: Statistical package for the social sciences
